# Lipid accumulation facilitates mitotic slippage-induced adaptation to anti-mitotic drug treatment

**DOI:** 10.1038/s41420-018-0127-5

**Published:** 2018-11-27

**Authors:** Alex Wong, Sixun Chen, Lay Kien Yang, Yoganathan Kanagasundaram, Karen Crasta

**Affiliations:** 1 Lee Kong Chian School of Medicine, Nanyang Technological University, Singapore, Singapore; 2 Institute for Health Technologies, Interdisciplinary Graduate School, Nanyang Technological University, Singapore, Singapore; 3 Institute of Molecular and Cell Biology, Agency for Science, Technology, and Research, Singapore, Singapore; 4 Bioinformatics Institute, Agency for Science, Technology, and Research, Singapore, Singapore; 5 School of Biological Sciences, Nanyang Technological University, Singapore, Singapore

## Abstract

Aberrant lipid accumulation is a hallmark of cancer known to contribute to its aggressiveness and malignancy. Emerging studies have demonstrated context-dependent changes in lipid metabolism during chemotherapy. However, there is little known regarding the mechanisms linking lipid metabolism to chemotherapy-induced cell fates. Here, we describe lipid accumulation in cells following antimitotic drug treatment. Cells arrested in mitosis, as well as cells that escaped mitotic arrest and underwent mitotic slippage, showed elevated cytoplasmic lipid droplets. Interestingly, we found that TOFA, a lipid biosynthesis inhibitor that targets acetyl-CoA carboxylase (ACC) and blocks lipid accumulation, promoted early slippage, reduced cellular stress and enhanced survival of antimitotic-treated cells. Our work previously revealed that cells that survive after mitotic slippage can become senescent and confer pro-tumourigenic effects through paracrine signalling. Modulating lipid biosynthesis in cells post slippage by TOFA amplified their inflammatory secretion profiles and accelerated the development of tumourigenic behaviour, particularly cell migration and invasion, in a paracrine-dependent manner. In contrast to TOFA, inhibition of lipid accumulation by C75, a drug targeting fatty acid synthase (FASN), significantly reduced the production of pro-tumourigenic factors and associated phenotypic effects. This suggests that discrete lipid biosynthesis pathways could contribute differentially to the regulation of pro-tumourigenic inflammation. The divergent effects of TOFA and C75 may be attributed to the opposing regulation of Malonyl-CoA, an intermediate in fatty acid synthesis that serves as a mediator of fatty acid oxidation. Taken together, our data reveal a previously unappreciated role for lipid accumulation in the cellular adaptation to antimitotic drug treatment. Targeting lipid biosynthesis in cells post slippage may reprogramme its secretory profile such that it not only negates tumour-promoting effects, but may also promote anti-tumour inflammation for clearance of post-slippage senescent cells.

## Introduction

Antimitotic drugs, such as paclitaxel and vinblastine, are often used as first-line therapy against a broad range of cancers^1,2^. By targeting microtubule dynamics, these drugs affect cell proliferation culminating in a mitotic arrest and eventually mitotic cell death. However, cells could also take an alternative cell fate route known as mitotic slippage, a process where cells exit mitosis and enter interphase without going through proper chromosome segregation and cytokinesis^3^. As a result, cells post slippage tend to be tetraploid and multinucleated. Previous studies have described various cell fates post slippage including: (1) apoptosis, (2) cell cycle arrest that culminates in senescence and (3) proliferation as genomically unstable cells^4^.

While several mechanistic studies have alluded to cell death post slippage^5,6^, there has been little describing molecular pathways leading to cell cycle arrest and the ensuing senescence post slippage. We have previously shown that the senescence-associated secretory phenotype (SASP) factors^7^, consisting of various cytokines, chemokines and growth factors released by post-slippage senescent cells, promote tumourigenic behaviour in neighbouring cells^8^. Persistence of cells post slippage may undermine the effectiveness of antimitotic drugs and ultimately contribute to the development of tumour recurrence and chemoresistance. Hence, it is crucial to gain better mechanistic understanding of the senescent cell fate post slippage for enhanced therapeutic strategies involving the elimination of senescent cells or its associated pro-tumourigenic effects post slippage following antimitotic therapy.

Enhanced lipid biosynthesis is a characteristic feature of cancers. Indeed, aberrant lipid accumulation in cancer cells has emerged as a possible diagnostic and therapeutic target^9^. In cancer cells, the supply of cellular fatty acids is highly dependent on the de novo fatty acid synthesis^10^. This involves two key enzymes, acetyl-CoA carboxylase (ACC) and fatty acid synthase (FASN). ACC carboxylates acetyl-Co to form malonyl-CoA. The malonyl-CoA is further converted to long-chain fatty acids by FASN. Acyl-CoA synthetase then coverts fatty acid to acyl-CoA.

Chemotherapeutic drugs doxorubicin and 5-fluorouracil that are used in the treatment of human colorectal and breast cancer cells have previously been reported to induce the accumulation of cytoplasmic lipid droplets (LDs)^11–13^. Additionally, LD induction during apoptosis in murine lymphoma cells treated with etoposide has been shown to result from inhibition of mitochondrial fatty acid oxidation, in which fatty acids are directed towards the de novo fatty acid synthesis^14^. A similar mechanism governing LD accumulation was described in neuroblastoma cells treated with a c-Myc/Max inhibitor^15^. Ceramide metabolism has also been implicated as a key regulator of sensitivity to paclitaxel and other chemotherapeutic drugs^16^. Importantly, in addition to apoptotic cells, a role for LD accumulation has also been observed in senescent cells as well. Senescent cells have been shown to contain LDs that are more numerous and larger in size than their proliferating counterparts^17^. Murine melanoma cells incubated with delipidised media containing specific lipids such as ceramide and triglyceride significantly increased cellular senescence, suggesting that modulated lipid metabolism could contribute to the onset of senescence^17^.

Treatment with paclitaxel, the most commonly utilised antimitotic drug in the clinics, has been shown to induce LD accumulation following treatment^18^. Increased LD formation has been detected in both mitotically arrested and apoptotic cells, with the latter showing relatively higher accumulation. However, it is unknown if lipid biosynthesis influences cell fate following antimitotic drug treatment, particularly implications for mitotic slippage. Here, we describe a role for aberrant lipid metabolism during both mitotic arrest and post slippage following treatment with the antimitotic drug Nocodazole (Noc). We show that 5-(tetradecyloxy)-2-fuoric acid (TOFA), a lipid biosynthesis inhibitor that targets ACC and blocks lipid accumulation, promoted early slippage. The ensuing cellular stress was reduced, which in turn increased survival of antimitotic-treated cells. TOFA treatment also increased production of secretory factors that potentiated tumourigenic phenotypes, such as enhanced migration and invasion post slippage. Notably, senescence induction per se was not perturbed by lipid inhibition. Intriguingly, unlike TOFA-induced lipid inhibition, inhibition by C75 that targets FASN (an enzyme directly downstream of ACC) drastically reduced the production of inflammatory secretory factors in post-slippage cells via modulation of the NF-κB (nuclear factor kappa-light-chain-enhancer of activated B cells) pathway. This conundrum points to specific and differential inhibition of the de novo fatty acid synthesis pathway via inhibitors used. Therefore, it is plausible that discrete lipid biosynthetic pathways contribute differentially to the regulation of pro-tumourigenic inflammation post slippage. Taken together, our results reveal a previously unappreciated consequence of lipid accumulation in the regulation of mitotic cell fate, particularly mitotic slippage, and its associated pro-tumourigenic inflammation in response to treatment with antimitotic drugs.

## Results

### Antimitotic drugs induce lipid accumulation during mitotic arrest and after slippage

**Fig. 1:**
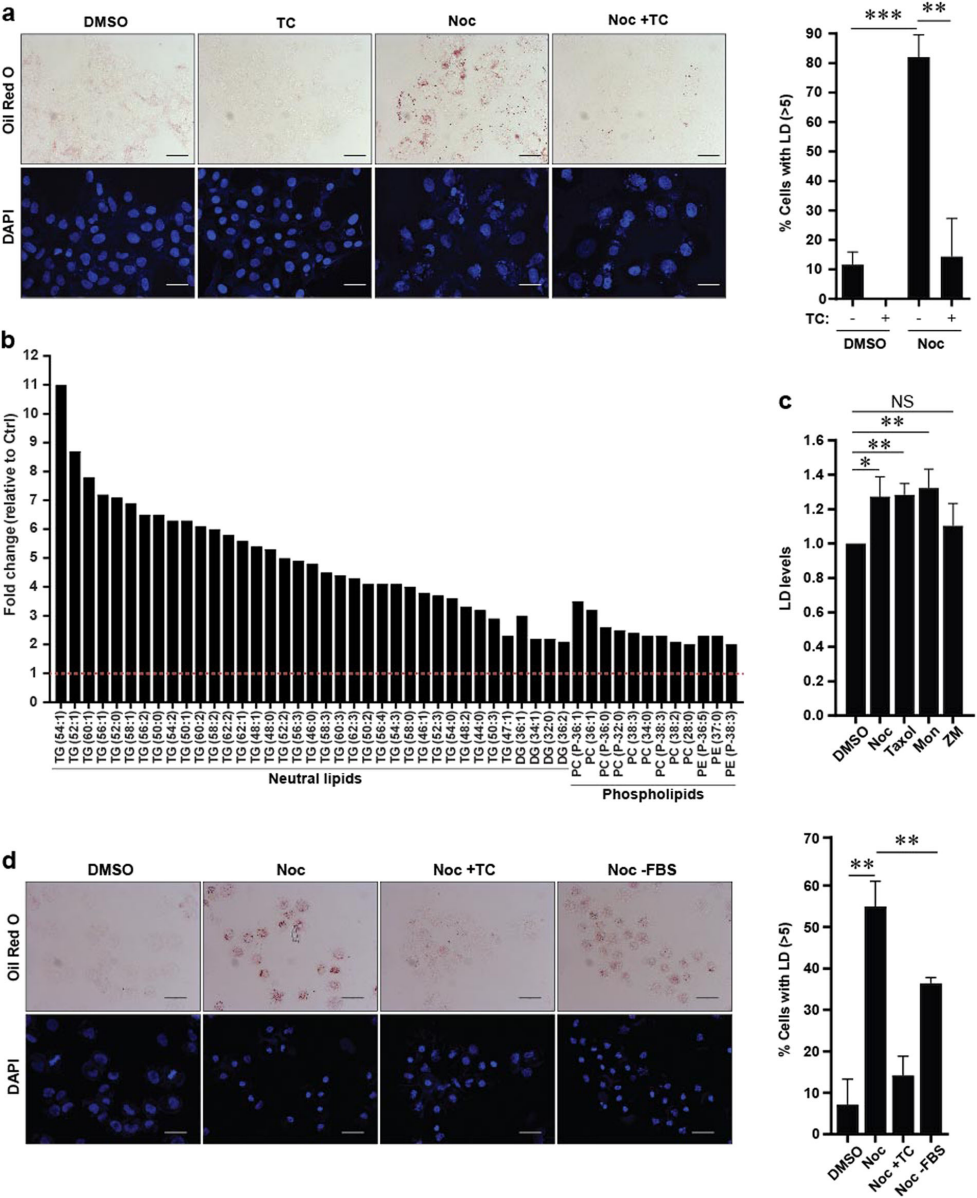
Lipid accumulation is induced in mitotically arrested and cells post slippage. **a** Left: Representative images of U2OS cells treated with DMSO or Noc for 48 h in the presence or absence of Triascin C (TC). Cells were stained with Oil Red O. Scale bar: 50  μm. Right: Quantification of cells with >5 LDs from (**a**). Data are mean ± s.d. of 3 independent experiments. **b** U2OS cells treated with Noc for 48 h were collected for lipidomic analysis. Each class of lipids was normalised with respective internal standards. Lipids identified were matched by Lipid Maps and confirmed with MS/MS. Lipid profiles shown are greater than 2-fold change (*p* > 0.05) with respect to DMSO-treated (Ctrl) cells. **c** Flow cytometric analysis of U2OS cells treated with different classes of antimitotic drugs for 48 h and analysed using BODIPY 493/503 staining. LD levels represent the mean fluorescence intensity values of the total cell population for each sample. Data are mean ± s.d. of 3 independent experiments. **d** Left: Representative images of U2OS cells treated with the indicated drugs or inhibitors for 24 h and stained for Oil Red O (red) and DAPI (blue). Scale bar: 50 µm. Right: Quantification of cells with >5 LDs from (**d**). Data are mean ± s.d. of 3 independent experiments; *p < 0.05, **p < 0.01, ***p < 0.001 and NS non-significance by Student’s *t*-test

To interrogate the effects of antimitotic drugs in modulating lipid metabolism, we first treated mitotic slippage-prone osteosarcoma U2OS cells with Noc and assessed lipid accumulation in these cells. Consistent with our previous observations^8^, more than 80% of U2OS cells underwent mitotic slippage and displayed multinucleation, following Noc treatment for 48 h (Supplementary Fig. S1a). This was further confirmed by western blot profiles showing time-dependent degradation of mitotic markers cyclin B1, decreased phosphorylation of histone-H3 (pH-H3) and decreased levels of spindle assembly checkpoint marker BubR1 (Supplementary Fig. S1b). To assess lipid accumulation post slippage, U2OS cells were treated with Noc for 48 h and subsequently stained with Oil Red O, a dye that stains neutral triglycerides and LDs. An increase in cytoplasmic LDs was observed in U2OS cells post slippage compared to dimethyl sulfoxide (DMSO)-treated control cells (Fig. 1a). Triascin C (TC), an inhibitor of long fatty acyl-CoA synthetase (an essential enzyme for lipid droplet biosynthesis), significantly reduced LD accumulation in Noc-treated cells post slippage, confirming a bona fide lipid accumulation (Fig. 1a). LD accumulation was also observed in HCT116 human colon carcinoma and MDA-MB-231 breast adenocarcinoma cell lines post slippage following treatment with Noc for 48 h (Supplementary Fig. S2a, b).

To further validate lipid accumulation in cells post slippage, we performed lipidomic analyses of DMSO-treated and 48 h Noc-treated U2OS cells using mass spectrometry. Lipidomic profiles normalised to respective internal standards revealed a significant upregulation of lipid metabolites, particularly the lipid species triacylglycerol (TAGs), in the Noc-treated samples compared to control cells (Fig. 1b). TAGs are neutral lipid species that are well defined as the major composition found in LDs^19^. To test whether LD accumulation occurs under other antimitotic drug conditions that can also induce mitotic slippage after 48 h of treatments, we assayed for neutral lipid species using the fluorescent neutral lipid dye 4,4-difluoro-1,3,5,7,8-pentamethyl-4-bora-3a,4a-diaza-s-indacene (BODIPY 493/503). Flow cytometric detection of BODIPY 493/503 confirmed increased LD accumulation in cells post slippage induced by various classes of antimitotic drugs such as microtubule-stabilising Taxol, mitotic kinesin Eg5 inhibitor Monastrol and Aurora-B kinase inhibitor ZM447439 compared to DMSO-treated cells (Fig. 1c). ZM447439 overcomes the spindle assembly checkpoint, leading to an earlier exit from mitotic arrest and entry into mitotic slippage. It was observed that LD formation in ZM447439-treated cells was not significantly increased compared to control (Fig. 1c). This led us to ask whether arrest in mitosis was necessary for lipid accumulation post slippage. To this end, we treated cells with Noc for 24 h to enforce a prolonged mitotic arrest. We observed that approximately 56% of mitotically arrested cells were observed to contain LDs compared to DMSO-treated cells (8%) (Fig. 1d). In addition, we observed a modest reduction in the percentage of LD-abundant mitotically arrested cells when cultured in serum-depleted media (0% foetal bovine serum (FBS)) (Fig. 1d), indicating that exogenous lipid uptake may contribute to the elevated lipid content. Overall, our findings suggest that antimitotic drug-induced lipid accumulation first accumulates during mitotic arrest, and consequently following mitotic slippage.

### Fatty acid uptake contributes to lipid accumulation in cells post slippage

**Fig. 2:**
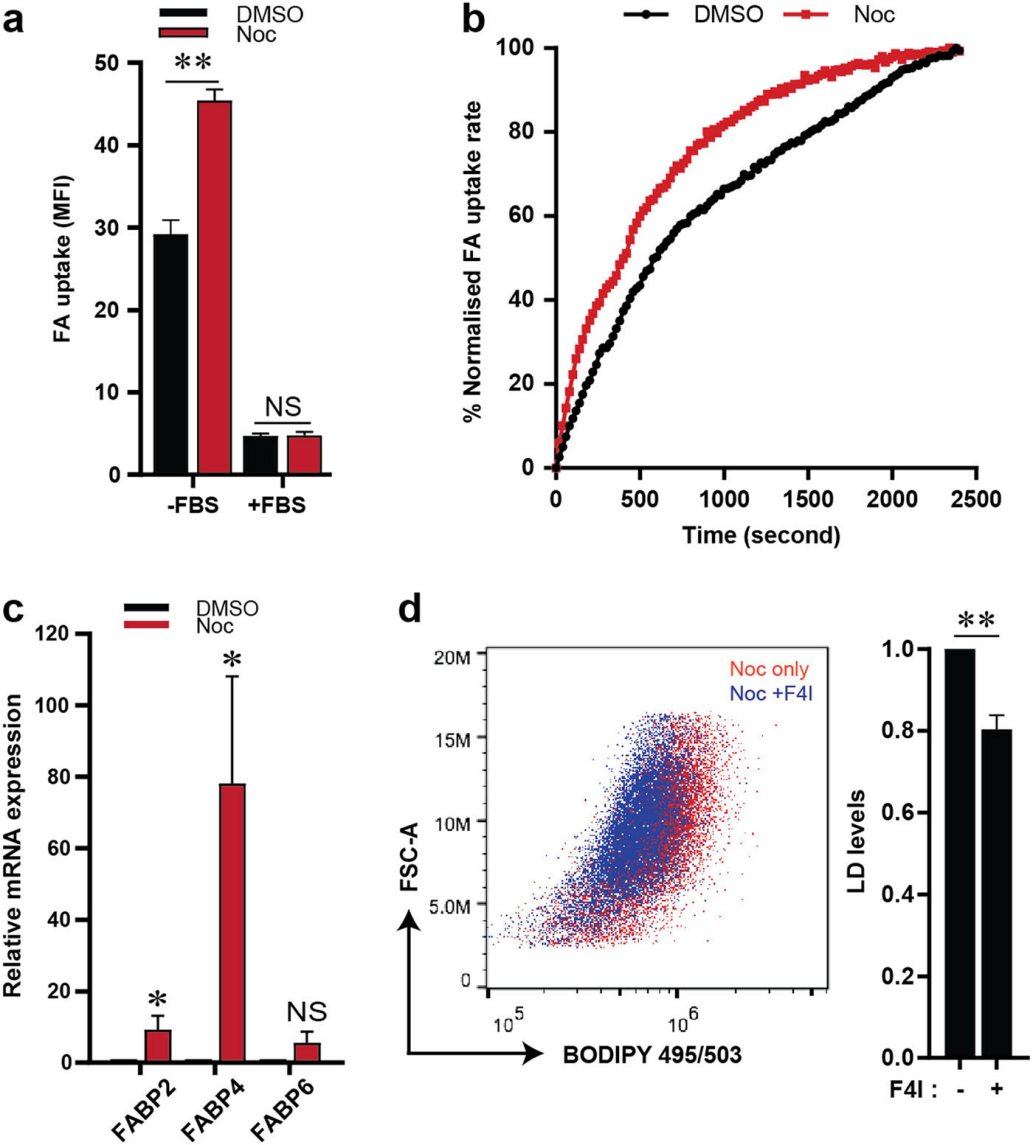
Enhanced fatty acid uptake contributes to LD accumulation. **a** FA uptake in U2OS cells treated with DMSO or Noc for 48 h. Serum starvation (SS) was performed after drug treatment to stimulate FA uptake. Negative control of FA uptake assay: normal serum (10% FBS) condition. Measurement of FA uptake in cells was quantified by mean fluorescence intensity (MFI). Data are mean ± s.d. of 3 independent experiments. **b** Representative plot of fatty acid uptake kinetics performed as per (**a**). **c** Synchronised U2OS were treated with DMSO or Noc for 48 h and analysed for FABP mRNA expression. **d** Flow cytometric analysis of U2OS cells treated with Noc alone or in combination with different concentrations of FABP4 inhibitor (F4I). Cells were stained for LDs using BODIPY 493/503. Data are mean ± s.d. of 3 independent experiments; *p < 0.05, **p < 0.01 and NS non-significance by Student’s *t*-test

The accumulation of lipids induced by anticancer drugs such as bevacizumab was shown to be due to an increased uptake of fatty acids (FAs) from the surrounding environment^20^. Since a slight reduction in LDs was observed in serum-depleted cells, we hypothesised that extracellular FA may contribute to lipid accumulation in our context as well. To assess the effect of Noc treatment on FA uptake in U2OS cells, we stimulated FA uptake by means of serum starvation where FBS addition into the culture media was omitted. Significant upregulation in the rate of FA uptake was observed in U2OS cells treated with Noc for 48 h compared to the DMSO-treated control (Fig. 2a, b). In support of this, microarray analysis of gene expression in Noc-induced post-slippage cells revealed a significant enrichment of fatty acid-binding protein 4 (FABP4) messenger RNA (mRNA) as compared to DMSO-treated cells (accession number: GSE114515) (Supplementary Table 1). Fatty acid-binding proteins (FABPs) belong to a family of cytoplasmic proteins known to be involved in FA uptake and transport^20,21^. We assessed the expression of FABPs by mRNA expression analysis which confirmed that FABP4 is significantly elevated in U2OS cells post slippage compared to control cells (Fig. 2c). To determine whether FABP4 could promote Noc-induced LD accumulation, we co-treated cells with Noc and a small molecule inhibitor of FABP4 (F4I), BMS309403. Inhibition of FABP4 led to a modest reduction in LD level in Noc-treated cells (Fig. 2d). Our findings suggest that FABP4 could mediate enhanced FA uptake that may partially contribute to LD accumulation in cells post slippage.

### Blocking acetyl-CoA carboxylase suppresses cellular stress and promotes survival of cells post slippage

**Fig. 3:**
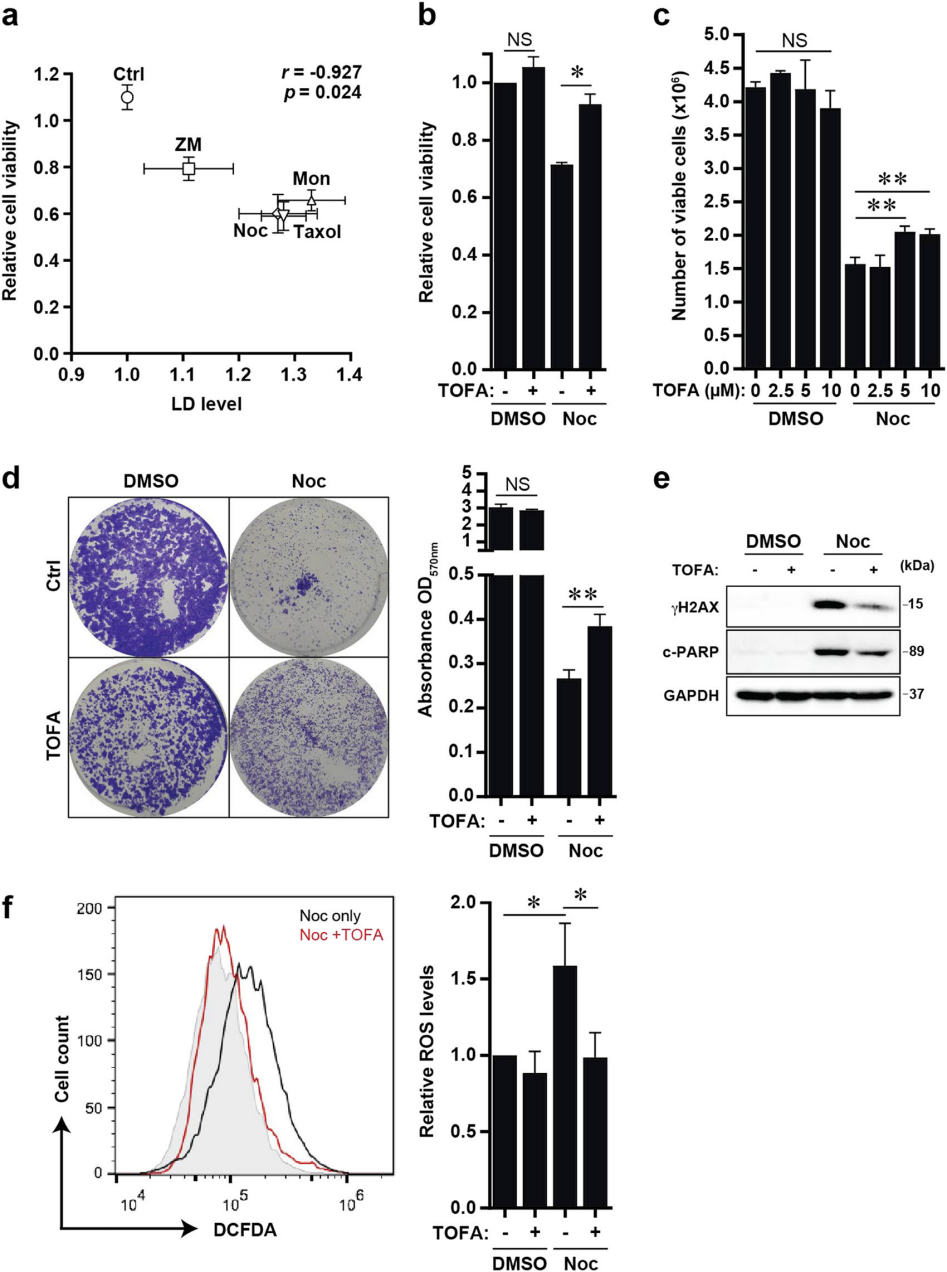
Lipid biosynthesis inhibitor TOFA suppresses cellular stress and promotes the survival of cells post slippage. **a** Cell viability and LD levels in U2OS cells after 48 h of treatment with respective antimitotic drugs. Cell viability was compared to respective LD levels and Pearson's correlation coefficients were determined. **b** Synchronised U2OS cells were treated with DMSO or Noc in the presence or absence of TOFA for 48 h, replated and grown for 24 h before cell viability assay was performed. Data are mean ± s.d. of 2 independent experiments. **c** Synchronised U2OS cells were treated with DMSO or Noc alone or in combination with TOFA for 48 h before quantifying the number of viable cells. Data are mean ± s.d. of 3 independent experiments. **d** Left: Long-term clonogenic assay of U2OS cells treated with DMSO or Noc in the presence or absence of TOFA. Cells were treated with respective drugs for 48 h, cultured in fresh media for another 5 days before crystal violet staining. Right: Plot shows the quantification of crystal violet staining. Data are mean ± s.d. of 3 independent experiments. **e** Western blot of U2OS cells treated with respective drugs for 48 h. **f** Left: Flow cytometry analysis of synchronised U2OS cells treated with respective drugs for 48 h, followed by DCFDA staining. Right: Quantification of ROS level in U2OS cells treated with respective drugs for 48 h. Data are mean ± s.d. of 3 independent experiments; *p < 0.05, **p < 0.01 and NS non-significance by Student’s *t*-test

It remains unclear as to whether LD accumulation upon antimitotic drug treatment is associated with cell survival or death. To ascertain this, we evaluated the cytotoxicity of various classes of antimitotic drugs described above (ZM447439, Monastrol, Nocodazole and Taxol) in U2OS cells. Relative cell viability was correlated to their respective cellular LD levels. Intriguingly, an inverse correlation between cell viability post treatment and LD levels was observed, suggesting that LD accumulation is associated with cell death (Fig. 3a). To further characterise the link between LD accumulation and antimitotic-induced cell death, we blocked lipid accumulation with a pharmacological inhibitor of lipid biosynthesis, TOFA. TOFA inhibits the rate-limiting enzyme of fatty acid biosynthesis pathways ACC which converts acetyl-CoA to malonyl-CoA (Supplementary Fig. 3a). Our results indicate that TOFA prominently reduced LD accumulation in Noc-induced cells post slippage (Supplementary Fig. 3b). Co-treatment of Noc and TOFA was able to further augment reduction of LDs and promote short- and long-term survival of cells post slippage (Fig. 3b–d). It is known that cells post slippage can acquire extensive DNA damage from prolonged mitotic arrest and multinucleation^5,22,23^. Therefore, TOFA treatment might alleviate antimitotic drug-induced stress response and promote cellular survival. Indeed, we observed that TOFA-treated cells post slippage displayed dramatic reduction in DNA damage (γ-H2AX levels) and reactive oxygen species (ROS) levels as compared to the LD-laden cells (Fig. 3e, f).

**Fig. 4:**
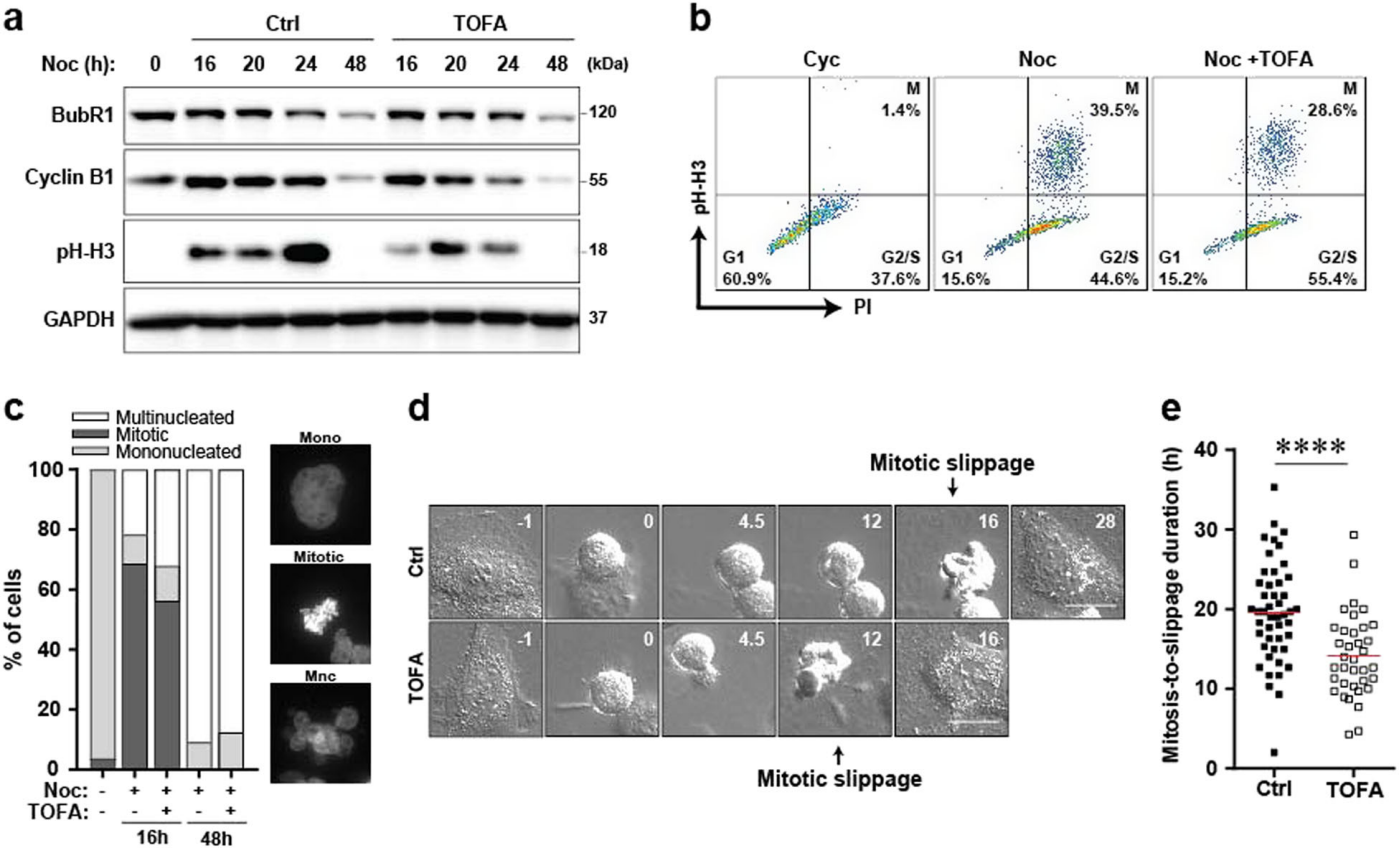
TOFA reduces the duration of Noc-induced mitotic arrest. **a** Synchronised U2OS cells were treated with Noc in the presence or absence of 10 μM TOFA for the indicated time period prior to immunoblotting analyses. **b** Flow cytometric analysis of mitotic cells stained using PI and FITC-pH-H3. Representative plots of U2OS cells treated with DMSO or Noc in the presence or absence of TOFA for 24 h. G1: G1 phase of the cell cycle, M: mitosis, G2/S: G2/S phase of the cell cycle. **c** Synchronised U2OS cells were treated with Noc in the presence or absence of 10 μM TOFA for the indicated time periods and stained with DAPI to visualise the nucleus. Left: Plot shows the percentage of cells that are mononucleated (Mono), mitotic or multinucleated (Mnc). Right: Representative images of cells that were quantitated. **d** Frames taken from the time-lapse of individual U2OS cells entering mitosis and subsequently undergoing mitotic slippage. Cells were treated with Noc in the presence or absence of TOFA. Numbers represent time (hours) relative to mitotic entry. Scale bar: 25 μm. **e** Plot is from a single representative experiment of the duration of mitotic arrest in U2OS cells treated with Noc in the presence or absence of TOFA. ****p<0001 by Student's *t*-test, n=47 without TOFA, n=36 with TOFA

Previous studies have shown that duration of mitotic arrest dictates the extent of multinucleation and DNA damage post slippage^22^. We hypothesised that lipid inhibition by TOFA may reduce the duration of mitotic arrest and promote early slippage upon Noc treatment. Our results demonstrate an increased degradation of cyclin B1, a trigger of mitotic exit^24^ and reduction in phosphorylated histone H3 levels when cells were co-treated with Noc and TOFA (Fig. 4a, b), suggesting earlier slippage. Consistent with this, we observed a modest increase in the percentage of multinucleated cells co-treated with TOFA at an early time point *t* = 16 h, compared to cells treated with Noc alone (Fig. 4c). To confirm this, we used live-cell imaging to discern the duration of mitotic arrest by measuring the time interval from cell rounding to mitotic slippage. A significant reduction in the duration of mitotic arrest was observed when cells were co-treated with TOFA and Noc (Fig. 4d, e). All the above suggest that blocking ACC in the lipid biosynthesis pathway with TOFA reduced the duration of Noc-induced mitotic arrest, which in turn decreased DNA damage and ROS levels in cells post slippage, possibly leading to their survival.

### Lipid biosynthesis differentially regulates post-slippage production of inflammatory factors and tumourigenic effects

**Fig. 5:**
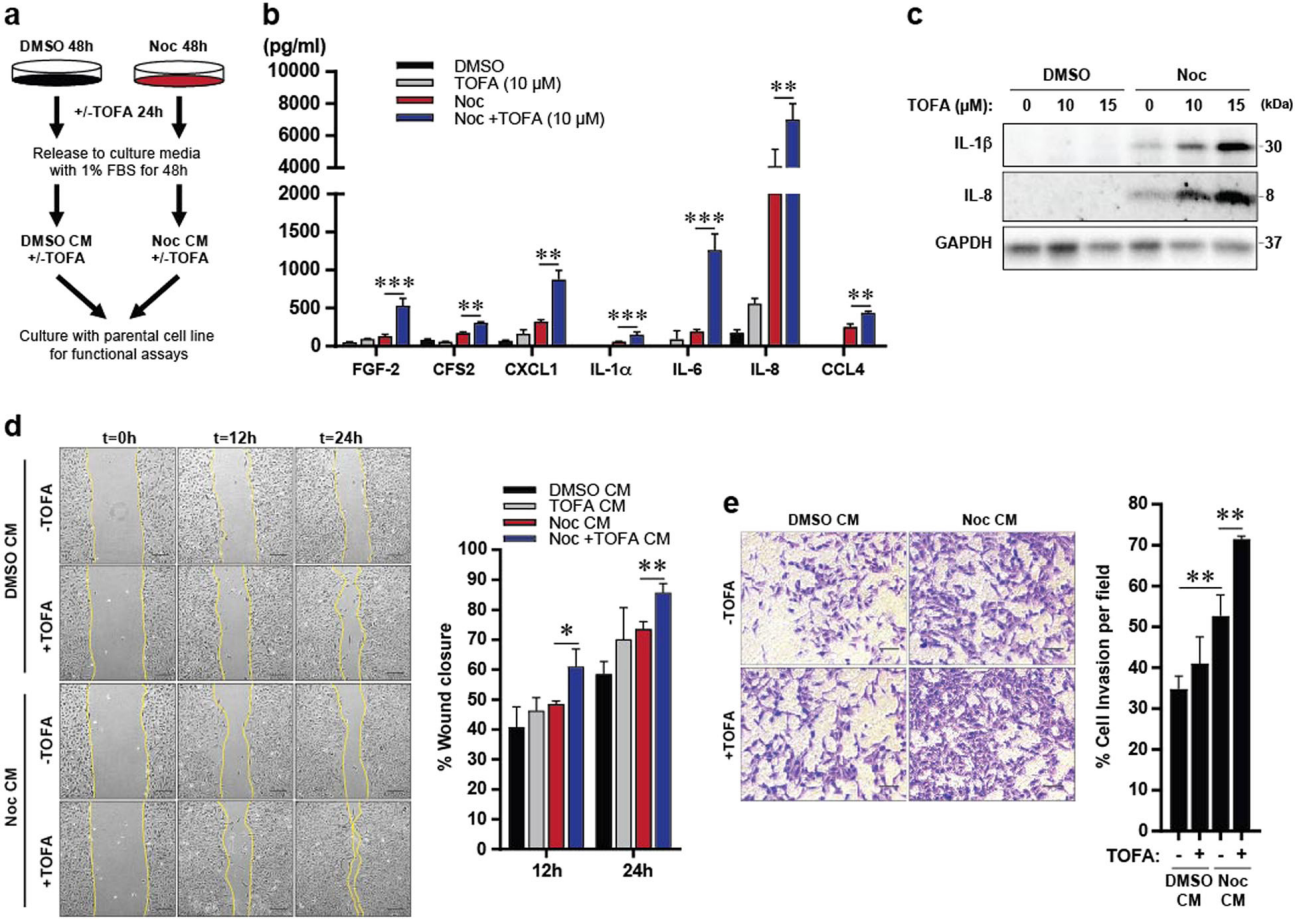
TOFA enhances SASP-induced migration and invasion post slippage. **a** Scheme depicts the preparation of respective conditioned media (CM) with or without TOFA addition. **b** Results from the human cytokine/chemokine array showing the concentration of various cytokines/chemokines in CM derived from respective treatments. Data are mean ± s.d. of 3 independent experiments. **c** Representative blots of synchronised U2OS cells treated with Noc for 48 h prior to treatment with various concentrations of TOFA for 24 h. **d** U2OS cells were incubated with the respective CM and subjected to the scratch wound healing assay. Left: Representative images of the assay taken at the indicated time intervals. Scale bar: 100 µm. Right: Plot showing percentage of wound closure. **e** U2OS cells were incubated with the respective CM and subjected to the transwell matrigel assay. Right: Representative images of invaded cells. Scale bar: 100 µm. Left: Plot showing percentage of invasive cells. Representative data from 3 independent experiments; *p < 0.05, **p < 0.01, ***p < 0.001 by Student’s *t*-test

We have previously shown that cells post slippage enter senescence, exhibit SASP and secrete pro-inflammatory factors such as interleukin (IL)-6, IL-8 and IL-1β which promote paracrine pro-tumourigenic effects such as migration, invasion and angiogenesis^8^. With regards to lipid metabolism, it has recently been revealed that therapy-induced senescent cells promote the build-up of lipids that potentially contribute to senescence induction^17^. To determine whether lipid biosynthesis regulates senescence following mitotic slippage, we treated U2OS cells with either Noc alone for 72 h or in combination with TOFA, and stained for the senescence-associated beta-galactosidase (SA-β-gal). Surprisingly, SA-β-gal staining revealed that there was no discernible difference in senescence establishment following mitotic slippage, with or without treatment with TOFA (Supplementary Fig. 4a). In addition, no discernable changes were observed in the expression of the senescence-associated Lamin B1 and p21 between control and TOFA-treated cells post slippage (Supplementary Fig. 4b), confirming that TOFA treatment did not affect post-slippage senescence induction. Interestingly, western blots also revealed augmented expression of the dominant cytokines detected post slippage as previously reported^8^, IL-8 and IL-1β, upon co-treatment of Noc and TOFA (Supplementary Fig. 4b). To exclude the possibility that the observed cytokine induction by TOFA treatment was due to early mitotic slippage, we subjected cells to 48 h of Noc treatment to allow them to enter a “post-slippage state”, followed by 24 h of treatment with TOFA. The conditioned media collected were analysed for secretion of pro-tumourigenic factors using enzyme-linked immunosorbent assay (ELISA)-based cytokine profiling (Fig. 5a). Consistent with our previous findings^8^, Noc-only treated cells showed increased secretion of several cytokines and chemokines such as IL-6, IL-8 and IL-1β (Fig. 5b). Lipid inhibition via treatment with TOFA in cells post slippage was observed to further augment secretion of these factors compared to control cells (Fig. 5b). Interestingly, increased expression of IL-8 and IL-1β correlated with a dose-dependent increase of TOFA, further supporting the notion that lipid inhibition increases inflammatory factors post slippage (Fig. 5c). Treatment of TOFA alone in U2OS cells did not induce the expression of IL-8 and IL-1β, suggesting that TOFA specifically regulates pro-inflammatory factors post slippage (Fig. 5c).

To assess whether TOFA affects the migratory and invasive properties induced by the factors secreted, we collected conditioned media (CM) from control or TOFA-treated cells post slippage for use in the scratch wound healing and transwell invasion assays. As shown in Fig. 5d, CM obtained from TOFA-treated cells post slippage increased the rate of wound closure compared to control cells, suggesting increased migration. Likewise, the transwell assay showed increased invasion of the bottom of the transwell chamber by cells incubated with CM from TOFA-treated post-slippage cells, suggesting increased invasion compared to controls (Fig. 5e). Overall, our results suggest that lipid inhibition by TOFA increased tumourigenic effects.

**Fig. 6:**
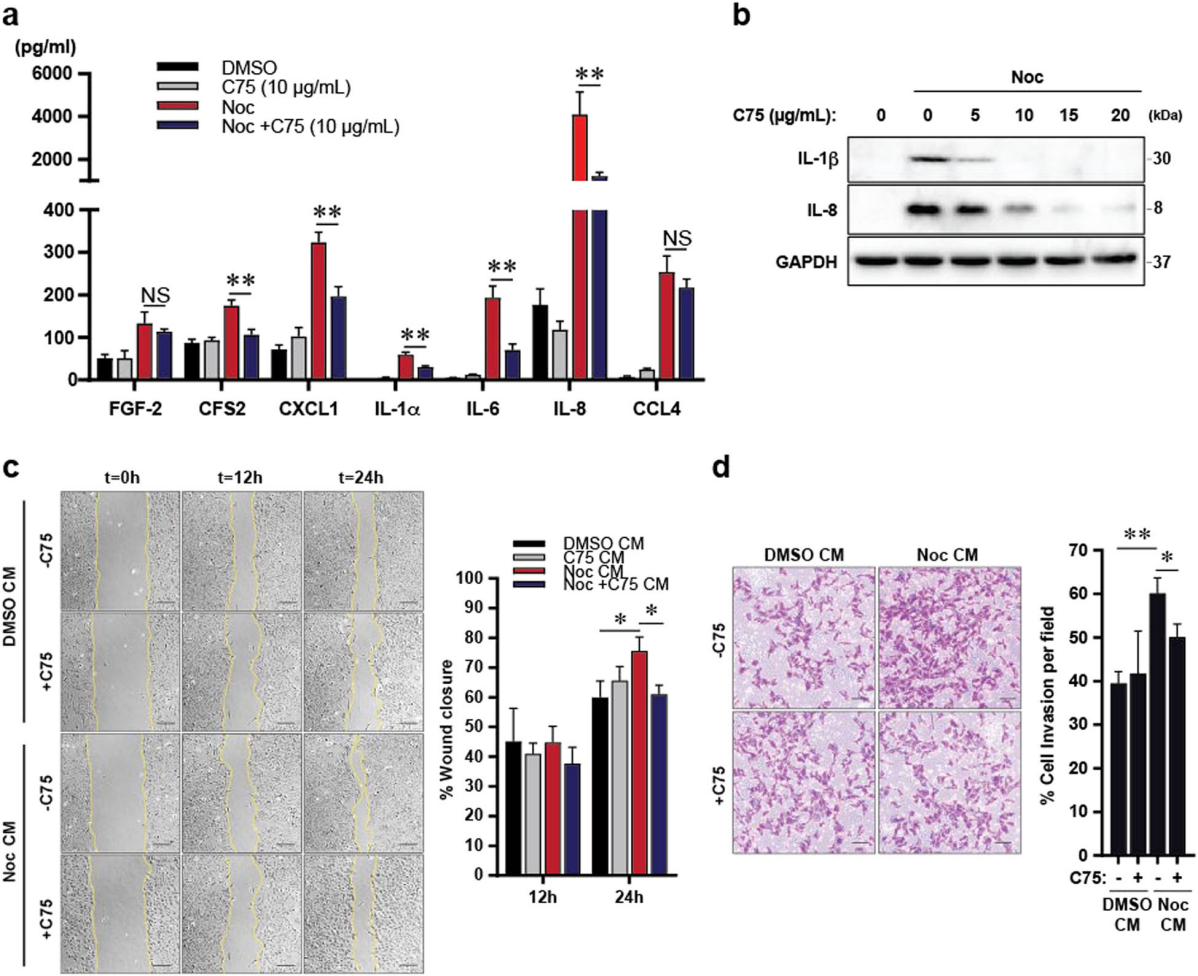
C75 abrogates motility and invasiveness induced by secretion from cells post slippage. **a** Results from the human cytokine/chemokine array showing the concentrations of various cytokines/chemokines in the CM derived from the respective treatments. Data are mean ± s.d. of 3 independent experiments. **b** Representative blots of synchronised U2OS cells treated with Noc for 48 h prior to treatment with various concentration of C75 for 24 h. **c** U2OS cells were incubated with their respective CM and subjected to the scratch wound healing assay. Left: Representative images of CM-treated U2OS cells taken at the indicated time intervals. Scale bar: 100 µm. Right: Plot shows percentage of wound closure. **d** U2OS cells were incubated with the respective CM and subjected to the transwell matrigel assay. Right: Representative images of the invaded cells. Scale bar: 100 µm. Left: Plot showing percentage of invasive cells. Representative data from 3 independent experiments; *p < 0.05, **p < 0.01 and NS non-significance by Student’s *t*-test

To further confirm the effect of lipid inhibition on inflammatory factors post slippage, we treated Noc-induced post-slippage cells with another inhibitor of lipid biosynthesis, C75, and analysed the expression and secretory profiles of several cytokines and chemokines. C75 inhibits FASN that is directly downstream of ACC (Supplementary Fig. 3a)^25^. Surprisingly, contrary to TOFA, co-treatment with C75 suppressed the secretion of several cytokines and chemokines such as IL-6, IL-8 and IL-1β (Fig. 6a, b). This finding was further supported by co-treatment with Orlistat, another inhibitor of FASN, that also suppressed the expression of IL-8 and IL-1β (Supplementary Fig. 5). In addition, CM derived from post-slippage cells co-treated with C75 attenuated the migratory and invasive capabilities compared to control (Fig. 6c, d). Our findings suggest that FASN inhibition by C75 could present a strategy to prevent tumourigenic effects following mitotic slippage. Our data suggest that targeting the fatty acid biosynthesis pathway at different nodal points may differentially regulate the production of inflammatory factors controlling tumourigenic action.

### FASN inhibition suppresses inflammatory cytokines by inhibiting NF-κB activity

**Fig. 7:**
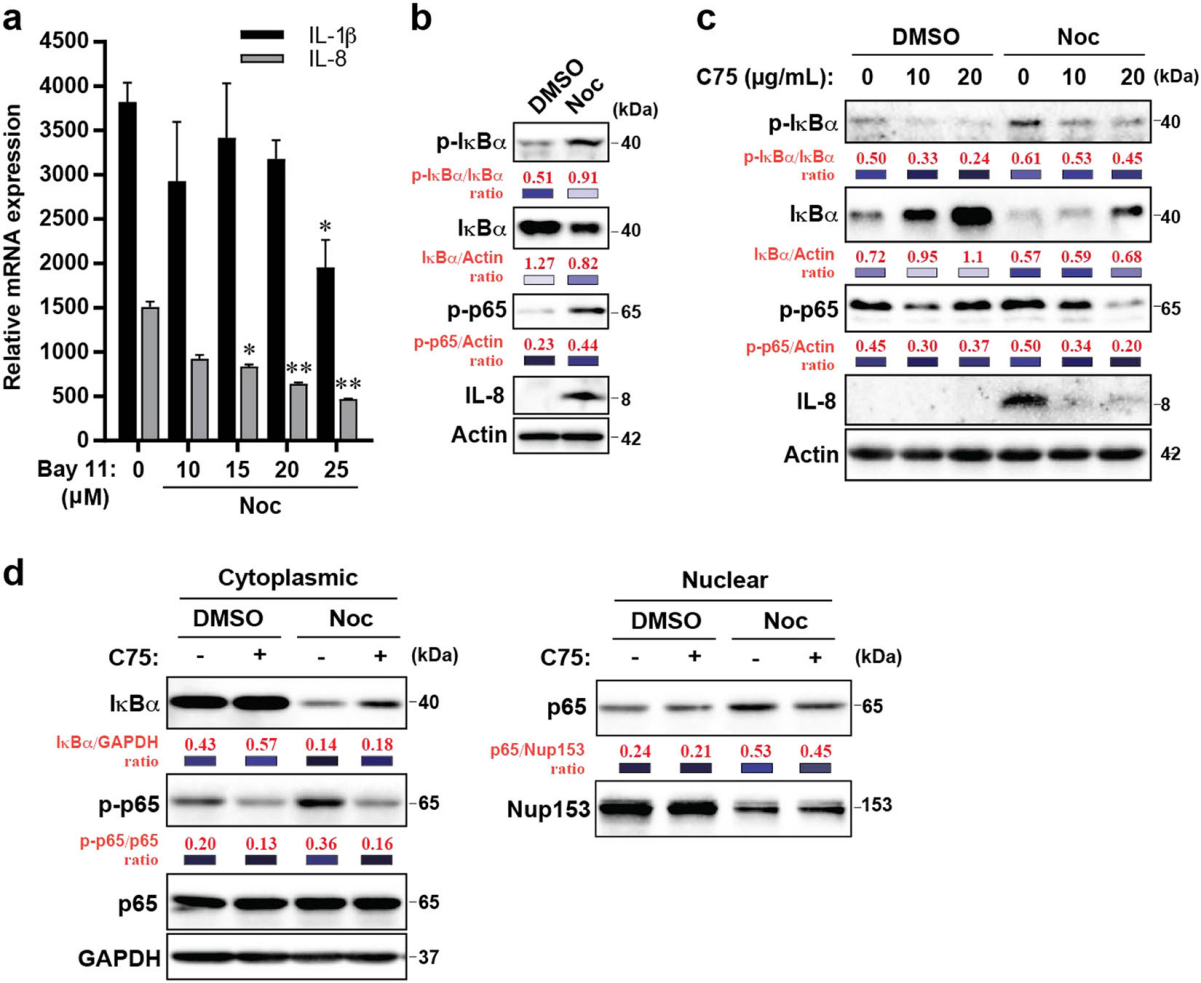
C75 inhibits Noc-induced NF-κB activation. **a** Synchronised U2OS cells were treated with Noc for 72 h, washed and incubated with various concentrations of Bay 11-7082 for 1.5 h. Cells were then collected for quantitative reverse transcription-PCR (qRT-PCR) analysis. The normalised data were expressed relative to the DMSO-treated (Ctrl) cells. Data from three independent experiments. *P*-values were derived from Student's *t*-test (*p < 0.05, **p < 0.01).  **b** Representative blots of synchronised U2OS cells treated with DMSO or Noc for 48 h and released into drug-free media for another 24 h. **c** Representative blots of synchronised U2OS cells treated with Noc for 48 h prior to treatment with various concentrations of C75 for 24 h. **d** Representative blots of synchronised U2OS cells treated with Noc for 48 h prior to treatment with 15 µg/mL of C75 for 24 h. Western blots show cytoplasmic and nuclear fractions of the cells. GAPDH and Nup153 function as loading markers for cytoplasmic and nuclear fractions respectively

The production of pro-tumourigenic cytokines such as IL-1, IL-6 and IL-8 are known to be induced by the NF-κB family of transcriptional factors^26^. Previous reports have also described activation of NF-κB-dependent inflammatory response upon paclitaxel treatment in multiple cancer cells^27–29^. Hence, one could predict that the suppression of pro-tumourigenic inflammation by FASN inhibitor C75 in cells following mitotic slippage could be due to the suppression of NF-κB activation. To test this, cells were co-treated with Noc and increasing concentrations of the NF-κB inhibitor Bay 11-7082. Results indicate that NF-κB inhibition resulted in the dose-dependent reduction of IL-8 and IL-1β expression, affirming that cytokine production upon Noc treatment is mediated through NF-κB pathway (Fig. 7a). Western blotting analysis revealed enhanced phosphorylation of IκBα and p65, suggesting that the NF-κB pathway is activated in cells post slippage (Fig. 7b). Interestingly, treatment of cells post slippage with the C75 inhibitor suppressed NF-κB activation as shown by reduced IκBα and p65 phosphorylation compared to DMSO-treated cells (Fig. 7c). This was further confirmed by the modest reduction of nuclear p65 in C75-treated cells compared to control (Fig. 7d). These results suggest that lipid inhibition by C75 suppressed the expression of inflammatory factors via NF-κB inactivation and subsequently perturbed the pro-tumorigenic effects induced by post-slippage cells.

## Discussion

**Fig. 8:**
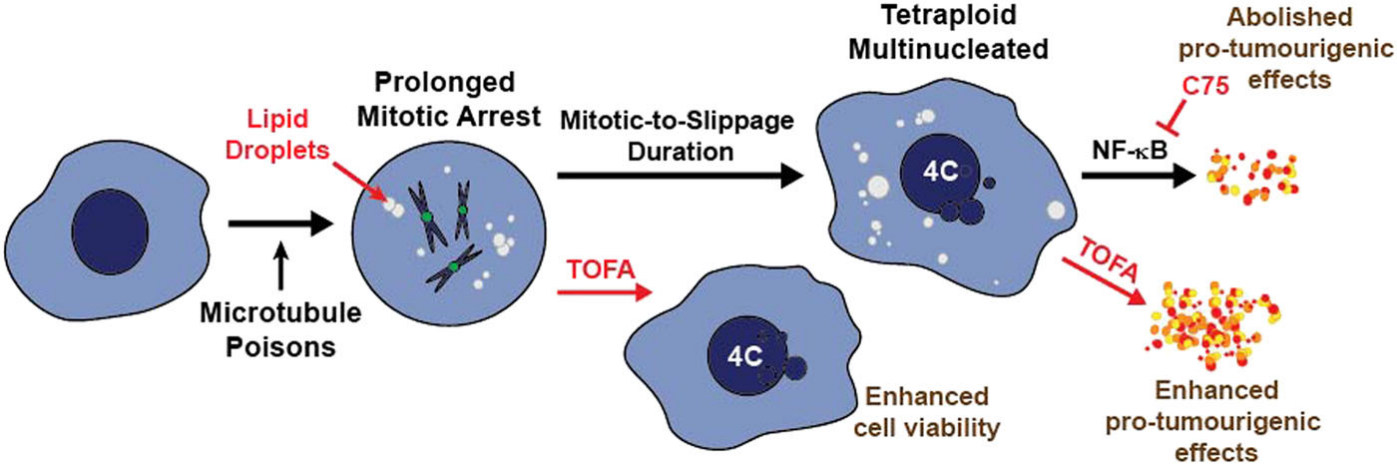
Proposed model depicting the role of lipid biosynthesis in the regulation of cell fate upon antimitotic drug treatment. Upon treatment with antimitotic drugs, mitotic-arrested cells accumulate lipid droplets (LDs) that persist during and after mitotic slippage. Co-treatment with acetyl-CoA carboxylase inhibitor TOFA and antimitotic drugs shorten the duration of mitotic arrest resulting in LD-depleted cells post slippage. Treatment of TOFA amplifies the secretion of tumour-promoting inflammatory factors and its associated effects, whereas treatment with C75, a fatty acid synthase inhibitor, shows an opposing phenomenon. Taken together, these indicate that there is differential regulation of the de novo fatty acid synthesis pathway in controlling phenotypic effects post slippage

Although many anticancer drug treatments induce lipid accumulation in cancer cells^11–13,15,17,18^, the consequences of this phenomenon in affecting the efficacy of anticancer drugs remain largely unknown. In this study, we explored the role of lipid metabolism in regulating cell fate upon treatment with antimitotic drugs. We had previously proposed mitotic slippage-induced senescence and SASP following antimitotic drug treatment to be an important determinant of cancer cell survival^8^. The findings presented here demonstrate pharmacological inhibitor-induced changes in lipid biosynthesis in cells treated with Noc and their implications for tumour-promoting effects of cells post slippage (Fig. 8). Intriguingly, we found that inhibition of lipid accumulation could promote early mitotic slippage and contribute to the survival of cells post slippage. Although the incidence of senescence per se was not affected, lipid inhibition could modulate production of secretory factors (SASP) post slippage in a NF-κB-dependent manner, contingent on the lipid biosynthetic pathway invoked. Our study highlights the potential of targeting lipid biosynthesis in cells post slippage to reprogramme its secretory profile in a manner that could not only negate tumour-promoting effects, but may also promote anti-tumour inflammation for clearance of tumour cells.

Lipid accumulation upon chemotherapy can be triggered by several mechanisms such as enhanced lipogenesis, increased uptake of lipids and inhibition of FA oxidation^30^. Our study demonstrated that the accumulation of lipid content in cells post slippage was partly contributed by an increase in FABP4-mediated FA uptake. FABP4 is known to be upregulated in many aggressive cancers as well as recurrent tumours to facilitate the uptake and transport of extracellular FA from adipocytes to mitochondria for β-oxidation^31–33^. More work will have to be done to delineate the role of FABP4 in mediating post-slippage cell fate. Lipid accumulation upon antimitotic drug treatment can also occur from the defective or loss of mitochondria during mitotic arrest^34^. As such, impairment of mitochondrial β-oxidation promotes LD formation by diverting FA towards storage in LDs^14,15^.

The duration of mitotic arrest is known to be tightly linked to the extent of multinucleation and DNA damage following mitotic slippage^5^. We did not observe any significant increase in LD levels in cells treated with ZM447439, an Aurora kinase inhibitor that shortens the duration of mitotic arrest by overcoming the spindle assembly checkpoint. Blocking lipid biosynthesis by TOFA promoted early mitotic slippage, suggesting a role for lipids in ensuring proper mitotic progression. Support for this can be garnered from the finding that small interfering RNA-mediated depletion of lipid biosynthesis enzymes such as DGAT2 (a TAG synthesising enzyme) resulted in defective cell division, reminiscent of mitotic slippage^35^. Incidentally, secreted factor(s) from tumour-associated macrophages (TAMs) have been found to suppress the cytotoxicity effects of antimitotic agents by promoting early mitotic slippage and cancer cell survival following paclitaxel treatment^36^. It will be of interest to investigate whether TAMs engender mitotic slippage via modulation of lipid biosynthetic pathways.

Several inflammatory signalling pathways have been proposed to be activated by multinucleated cells^37^. Our previous study had shown pro-tumorigenic inflammatory factors to be secreted by senescent cells post slippage^8^. Inhibition of autophagy abrogated the induction of senescence and its associated secretion of inflammatory factors. Although the inhibition of lipid biosynthesis did not affect the establishment of senescence post slippage, we observed an alteration in the secretory profile of cells. The inhibition of ACC and FASN by TOFA and C75 respectively differentially promoted opposing effects on the production of secretory factors post slippage and consequent tumour-promoting effects (Fig. 8). This disparity could result from the regulation of mitochondrial FA oxidation by the different lipid inhibitors used. FA oxidation is known to regulate the expression and secretion of cytokines in various cell types such as in oncogene-induced senescent cells, tumour-associated macrophages and myeloid-derived suppressor cells^38–41^. We speculate and hypothesise that TOFA treatment decreases the level of malonyl-CoA, thereby preventing inhibition of FA oxidation as per scheme on Supplementary Fig. 3a ^42^. On the contrary, inhibition of FASN by C75 may promote the accumulation of malonyl-CoA, thereby further suppressing FA oxidation and the subsequent production of pro-tumourigenic factors^43,44^. Further work will need to be performed to confirm this hypothesis.

Alternatively, pro-tumourigenic factors could be altered by FA levels and saturation. It has been shown that fatty acids induce the expression and secretion of inflammatory cytokines such as IL-6, IL-8 and IL-1β via activation of NF-κB pathway in normal and cancer cells^45–47^. Indeed, our data revealed that C75 treatment suppressed the activation of NF-κB in post-slippage cells which supports the notion that increased FASN promotes the induction of post-slippage secretory factors. Another plausible inflammatory pathway involved could be via lipid mediators such as prostaglandin that is produced through conserved lipid biosynthesis pathways involving specific enzymes such as ACC and FASN. Collectively, our data suggest that lipid biosynthesis is involved in the modulation of pro-tumourigenic factors induced by cells post slippage.

Apart from the enhanced cell migration and invasion induced by post-slippage secretory factors, a distinct inflammatory response could also influence immune cell infiltration and the subsequent clearance of cells post slippage^4^. Alternatively, the pro-tumourigenic cytokines secreted may also stimulate the polarisation of M1 macrophages to M2 TAMs that may further enhance the pro-tumourigenic properties of neighbouring cells^40^. Indeed, paclitaxel treatment induces the infiltration of TAMs into mammary adenocarcinoma and suppresses antitumor T-cell response^28^, which may invoke properties of senescence and SASP post slippage. More extensive work will be needed to determine the effect of antimitotic-induced pro-tumourigenic factors on immune cells present in the tumour microenvironment and whether lipid metabolism is involved in mediating these effects.

## Materials and methods

### Cell culture and reagents

Human osteosarcoma U2OS, colorectal carcinoma HCT116 and breast adenocarcinoma MDA-MB-231 cancer cell lines were purchased from American Type Culture Collection (ATCC). Cells were maintained in Dulbecco’s modified Eagle’s medium-high glucose (Gibco), 10% FBS (GemCell) and 100 units/mL penicillin. Chemicals used were as follows: 100 ng/μL Nocodazole (N3000) (US Biological), 150 nM Paclitaxel (Taxol) (P1792A) (US Biological), 3 mM Thymidine (T5290) (Sigma), 100 μM Monastrol (1305) and 2 μM ZM447439 (2458) (TOCRIS). Lipid biosynthesis inhibitors used were as follows: Triascin C (ab141888) (Abcam), TOFA (T6575), C75 (C5490) and Orlistat (O4139) (Sigma). For cell synchronisation, cells were subjected to 24 h of serum starvation (0% FBS) followed by 3 mM thymidine arrest (G1/S arrest). Cells were released from thymidine for 3 h before the addition of fresh media with respective drugs.

### Immunoblotting

Whole-cell lysates were prepared in Pierce RIPA Buffer with Halt^TM^ Protease & Phosphatase Inhibitor Cocktail (Thermo Scientific). Protein concentration was measured using Bradford assay (Bio-Rad). An appropriate volume of Laemmli sample buffer was added to equal amounts of total proteins followed by boiling for 5 min. Cells lysates were resolved by TRIS–glycine sodium dodecyl sulphate–polyacrylamide gel electrophoresis gels (Bio-Rad), transferred onto nitrocellulose membranes and the membranes then incubated with primary antibodies overnight at 4 °C. The immunoblots were subsequently washed and incubated with horseradish peroxidase-conjugated anti-mouse or -rabbit antibodies (Amersham Biosciences) for 1 h before detection by ChemiDoc MP system (Bio-Rad) using ECL substrate (Thermo Scientific). Antibodies used were: Abcam: Lamin B1 (ab65986); IκBα (ab7217), Nup153 (ab171074); Bethyl:BubR1 (A300-386A); Cell Signaling Technology (CST): cleaved caspase-3 (Asp 175) (9664S), cleaved PARP (Asp214) (9546), phospho-histone H3 (Ser10) (9701S), phospho-histone H2A.X (Ser139) (2577), phospho-NF-κB p65 (Ser536) (3033), NF-κB p65 (D14E12) (8242), phospho-IκBα (Ser32/36) (9246), p21 Waf1/Cip1 (12D1) (#2947); Santa Cruz: Actin (sc-47778), cyclin B1 (H-20) (sc-594), p53 (DO-1) (sc-126); Novus Biologicals: CXCL8/IL-8 (6217), IL-1 beta/IL-1F2 (8516).

### Oil Red O staining

A working concentration of Oil Red O solution was made by diluting a 3.5 mg/mL stock solution (O0625) (Sigma) (in 100% isopropanol) with distilled water (6:4 ratio by volume). This solution was incubated at room temperature for 30 min and filtered through 0.2 μm syringe filter unit before use. Fixed cells were incubated in 60% isopropanol for 1 min, dried at room temperature and incubated in Oil Red O staining solution for 1 min. Cells were rinsed with distilled water two to five times and counterstained with Hoechst 33342 before mounting in ProLong Gold Antifade (P36935) (Life Technologies). Bright field images were captured using Nikon High Speed Live Cell Microscope (C11578-22C) with 40× objective.

### BODIPY 493/503 staining

BODIPY 493/503 (D3922) (Thermo Scientific) was purchased from Life Technologies. Live cells were trypsinised and washed twice in phosphate-buffered saline (PBS) before incubating in 2 μg/mL BODIPY (in PBS) for 15 min at 37 °C. After staining, cells were washed twice in PBS and analysed on an Accuri C6 flow cytometer (BD Biosciences) under FL-1 channel. Data were analysed using FlowJo software.

### Fatty acid uptake assay

Noc-treated cells were seeded at 50,000 cells/100 μL in each well of a 96-well plate. Cells were incubated in serum-free media at 37 °C for 1 h. For measurement of fatty acid uptake, the Free fatty acid uptake kit (Abam ab176768) used. Fatty acid dye-loading solution was then added to cells and fluorescence intensities measured at 20 s interval for 60 min with a fluorescence microplate reader (Biotek Cytation 5) at Ex/Em = 485/515 nm using a bottom read mode.

### Cell viability assay

For relative cell viability, cells were seeded in 96-well plates and treated as per their respective duration. Cell viability was assessed using CellTiter96® One Solution Proliferation Assays kit (G3580) (Promega) according to the manufacturer’s instructions. For absolute cell viability, cells were seeded in 10 cm dishes and treated as per their respective duration. Cells were then trypsined, stained with 0.4% Trypan blue solution and counted using an automated cell counter (Luna™). The number of viable cells = Total number of cells – number of blue cells.

### Clonogenic survival assay

U2OS cells were seeded at 7500 cells per well in 6-well plates. Cells were synchronised before the addition of respective drugs for 48 h. The media were then removed, cells were washed twice and fresh media were added. Plates were incubated for 5 days until macroscopic colonies formed. For quantification of colonies, the cells were washed twice with ice-cold PBS and fixed for 10 min with 4% paraformaldehyde (PFA). Colonies were stained with a 0.4% crystal violet/20% methanol solution for 10 min. The cells were then washed with water to remove excess dye, and dried at room temperature overnight. Colonies were quantified by solubilisation in 100% methanol followed by spectrophotometric analysis at 570 nm. The survival of treated cells was normalised relative to DMSO-treated cells. Statistical significance was determined by two-tailed Student’s *t*-tests.

### Cellular ROS measurement

Cells were trypsinised and washed twice in PBS before incubating in 20 μM of 2’,7’–dichlorofluorescin diacetate (DCFDA) (ab113851) (Abcam) working solution for 25 min at 37 °C. After staining, cells were washed in PBS and analysed on an Accuri C6 flow cytometer (BD Biosciences) under FL-1 channel. Data were analysed using FlowJo software. Fluorescence intensities were normalised against DMSO-treated cells.

### Preparation of conditioned media

Synchronised cells were treated with respective drugs for 48 h, followed by culture in drug-free media containing 1% FBS for additional 48 h. After collection, culture media were centrifuged at 5000 × *g*, filtered through a 0.22 μm pore filter (Pall Corporation) and mixed with media containing 40% FBS in a proportion of 3:1 to generate CM containing 10% FBS.

### Human cytokine and chemokine array

A total of 150 μL of CM was collected per condition for the assay. Then, 64 cytokine/chemokine/growth factor biomarkers were quantified simultaneously by using a Discovery Assay® called the Human High Sensitivity T-Cell Discovery Array 64-Plex (Eve Technologies Corp, Calgary, AB, Canada). The multiplex assay was performed at Eve Technologies by using the Bio-Plex™ 200 system (Bio-Rad Laboratories, Inc., Hercules, CA, USA), and a Milliplex Human High Sensitivity T-Cell panel (Millipore, St. Charles, MO, USA) according to their protocol. The 64-plex consisted of EGF, Eotaxin, FGF-2, Flt-3 ligand, Fractalkine, G-CSF, GM-CSF, GRO, IFN-α2, IFN-γ, IL-10, IL-12 (p40), IL-12 (p70), IL-13, IL-15, IL-17A, IL-1ra, IL-1α, IL-1β, IL-2, IL-3, IL-4, IL-5, IL-6, IL-7, IL-8, IL-9, IP-10, MCP-1, MCP-3, MDC (CCL22), MIP-1α, MIP-1β, PDGF-AA, PDGF-AB/BB, RANTES, TGFα, TNF-α, TNF-β, VEGF, sCD40L, Eotaxin-2, MCP-2, BCA-1, MCP-4, I-309, IL-16, TARC, 6CKine, Eotaxin-3, LIF, TPO, SCF, TSLP, IL-33, IL-20, IL-21, IL-23, TRAIL, CTACK, SDF-1a+B, ENA-78, MIP-1d and IL-28A. The assay sensitivities of these markers range from 0.11 to 3.25 pg/mL. The concentration of individual cytokines was determined based on the standard curve plotted from the determined concentration of a set standard and median fluorescent Intensity value.

### Cell migration and invasion assays

For scratch wound migration assay, cells were seeded in 6-well plates to 90% confluency. A p200 pipet tip was used to create a scratch on the cell monolayer. Cells were incubated with respective CM and the wound closure rate was tracked. For the cell invasion assay, cells were incubated with CM and plated on the top surface of transwell filter chambers pre-coated or uncoated with Matrigel (BD Biosciences). After 24 h of incubation, non-invasive cells on the top chamber were removed by swapping the surface with cotton buds and washed twice with PBS. Invaded cells at the bottom chamber were fixed with 4% PFA and stained with 0.05% crystal violet. The percentage of invasive cells was quantified by cell counting.

### Senescence-associated β-galactosidase staining

Cells were stained using the SA-β-gal Staining Kit (#9860) (CST) according to the manufacturer’s instructions.

### Time-lapse imaging

Cells were seeded in 6-well MatTek glass bottom dish at 4 × 10^5^ per well in a volume of 1.5 mL culture media. Synchronised cells were then washed with 1× PBS and released in 1.5 mL fresh media before addition of the respective drugs. Imaging was performed using Nikon High Speed Live Cell Microscope (C11578-22C) with 40× objective, with image collected every 15 min using 25 ms and 50 ms exposures for differential interference contrast (DIC) and green fluorescent protein (GFP) channels respectively. Captured image sequences were viewed and analysed using MetaMorph software.

### Lipidomic analysis by mass spectrometry

Six control cell samples (DMSO-treated) and six Nocodazole-treated cell samples (3 × 10^6^ cells each) were freeze-dried to obtain the dry weight. Cells were extracted with a methanol/dichloromethane (DCM)/water (1:2:0.2) mixture. Water was subsequently added to obtain a lower phase DCM layer enriched with lipids. Dried lipid extracts were then reconstituted in 300 µL of methanol. A quality control (QC) sample was created by combining 10 µl aliquot from each extract. A dilution series of the QC sample and IS (internal standard) were first analysed to ensure the MS responses were linear over the concentration range of the analytes. The 12 lipid extracts and QC samples were diluted to appropriate concentration and then mixed with pre-determined concentration of IS which contains 14 deuterated standards representative for 14 lipid classes. The extracts and QC sample spiked with IS were then analysed in triplicates in randomised order. Data were acquired in full-scan mode in both positive and negative ionisation modes. A separate high-resolution mass spectrometry (HRMS)/MS data were acquired on the QC sample using “auto MS/MS” mode for the identification of the lipid species.

## Electronic supplementary material

Supplementary Fig S1

Supplementary Fig S2

Supplementary Fig S3

Supplementary Fig S4

Supplementary Fig S5

Supplementary Table 1
