# Supplementary figures and images for: Lipid accumulation facilitates mitotic slippage-induced adaptation to anti-mitotic drug treatment
Source: Cell Death Discov. 2018 Nov 27;4:109. doi: 10.1038/s41420-018-0127-5 (PMC6258763; doi:10.1038/s41420-018-0127-5)

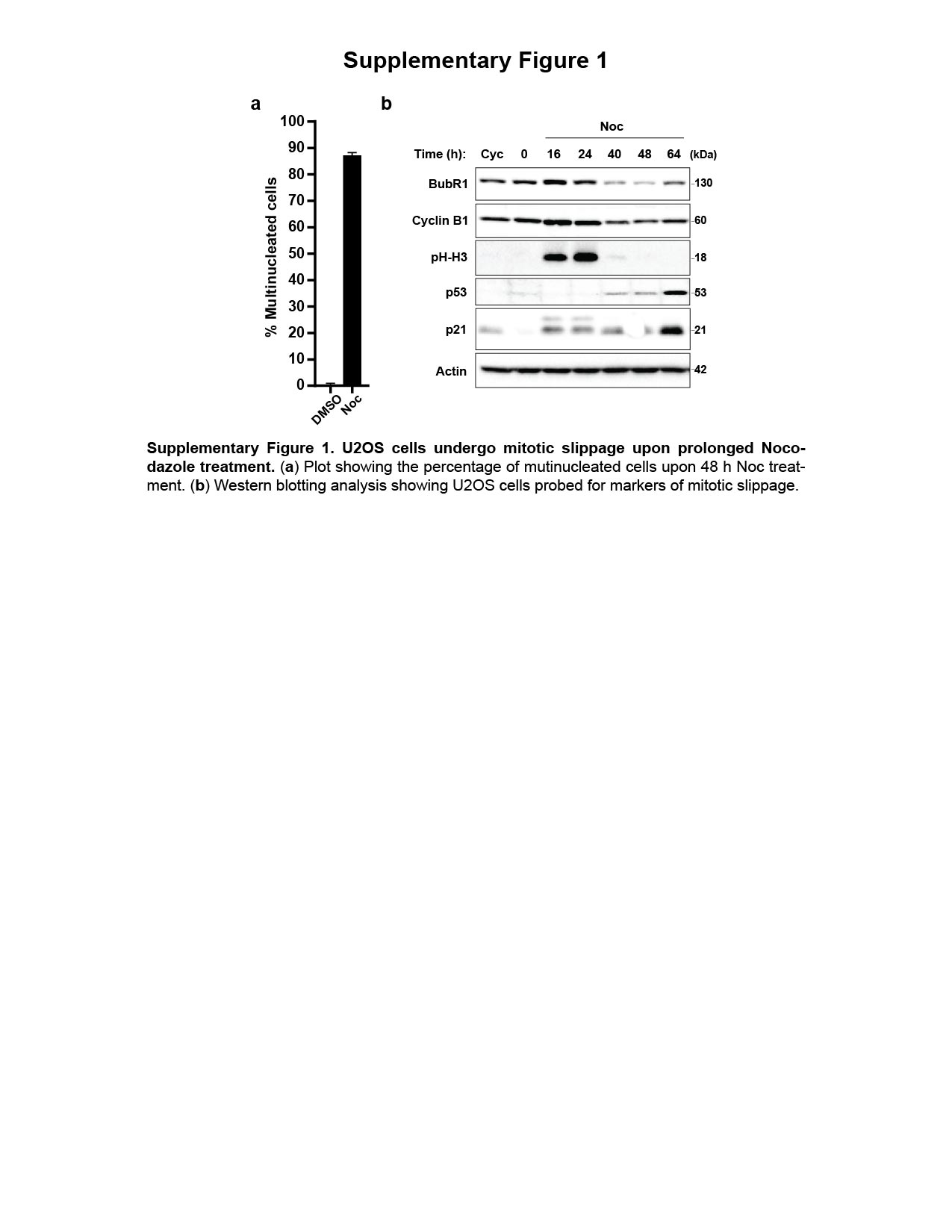

Supplement: Supplementary file 1 — Supplementary Fig S1 [file 41420_2018_127_MOESM1_ESM.jpg]

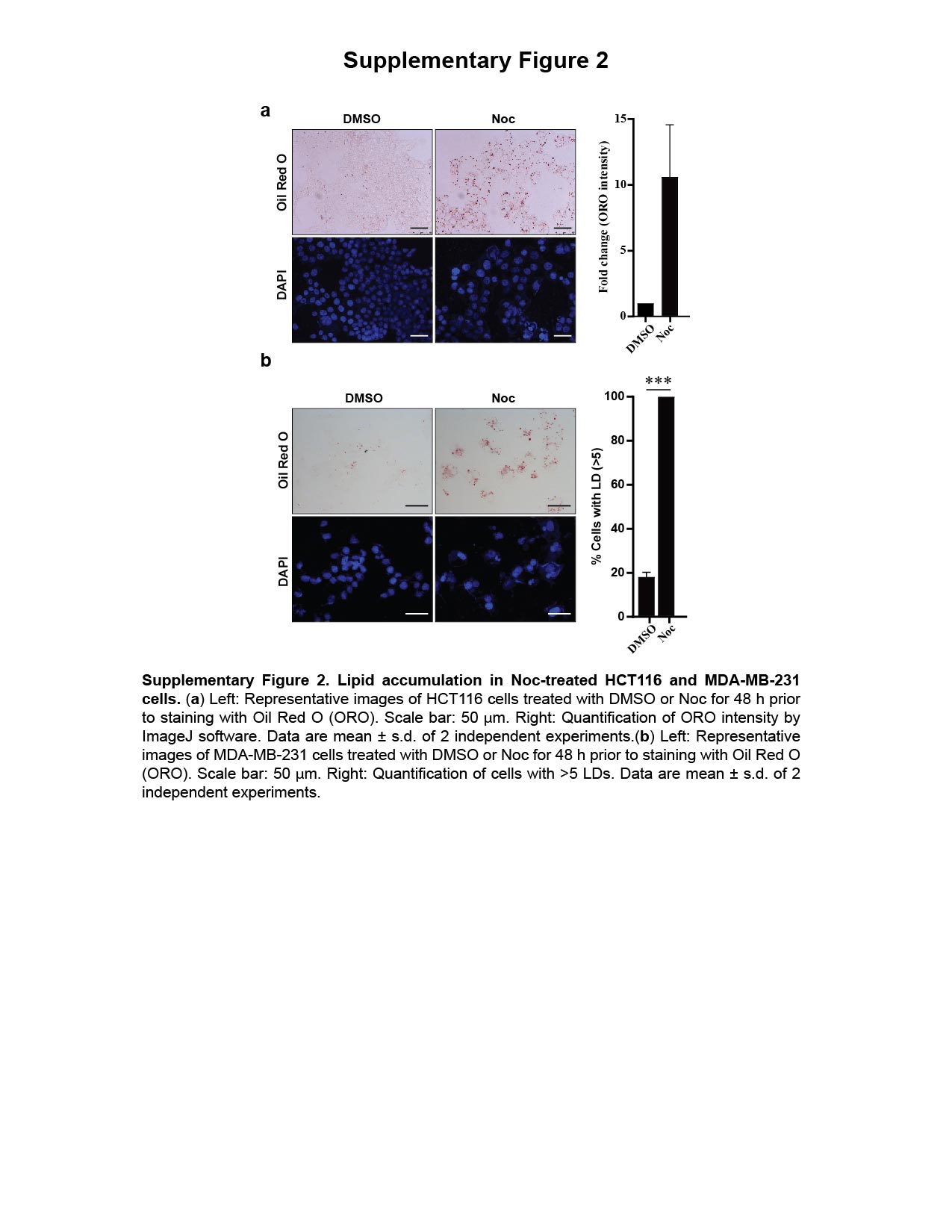

Supplement: Supplementary file 2 — Supplementary Fig S2 [file 41420_2018_127_MOESM2_ESM.jpg]

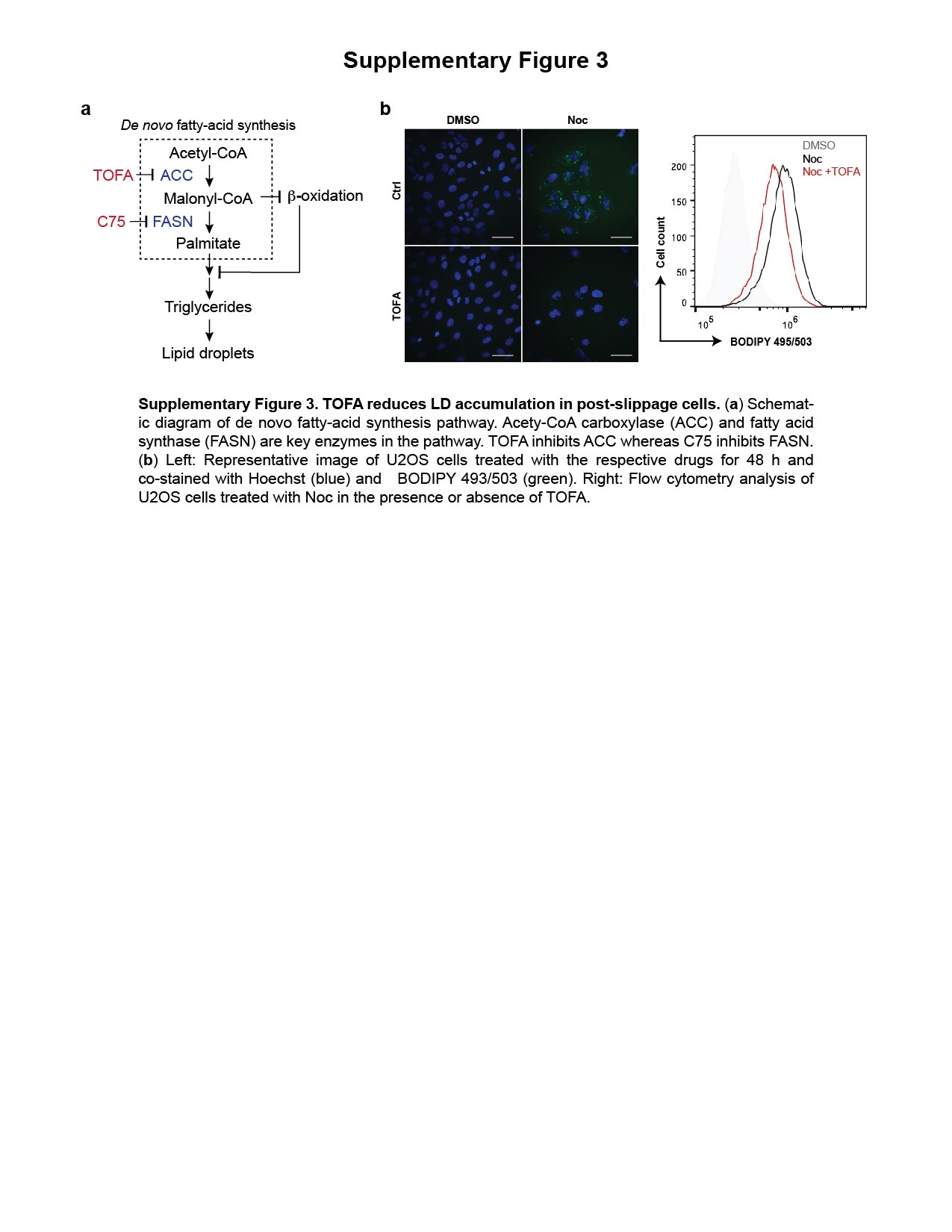

Supplement: Supplementary file 3 — Supplementary Fig S3 [file 41420_2018_127_MOESM3_ESM.jpg]

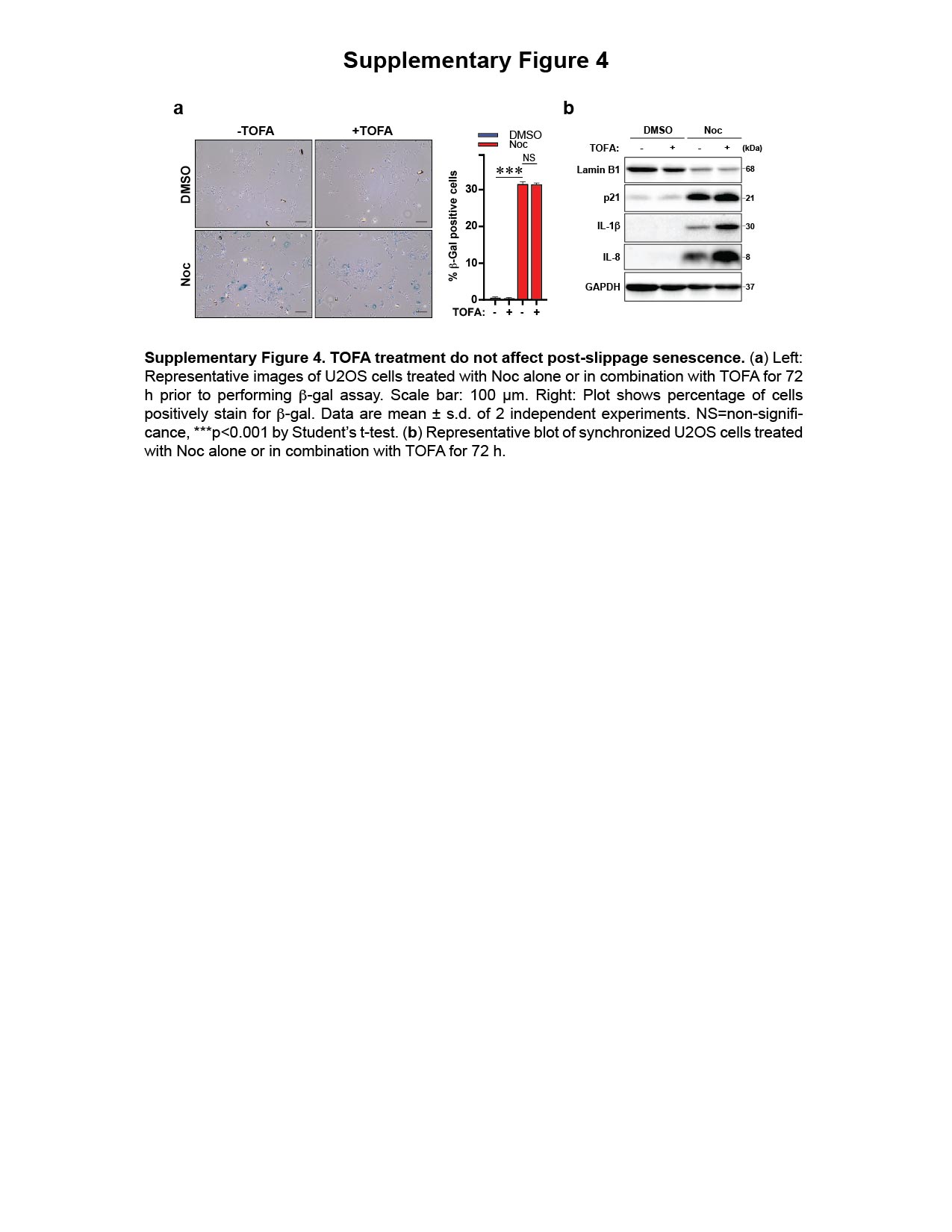

Supplement: Supplementary file 4 — Supplementary Fig S4 [file 41420_2018_127_MOESM4_ESM.jpg]

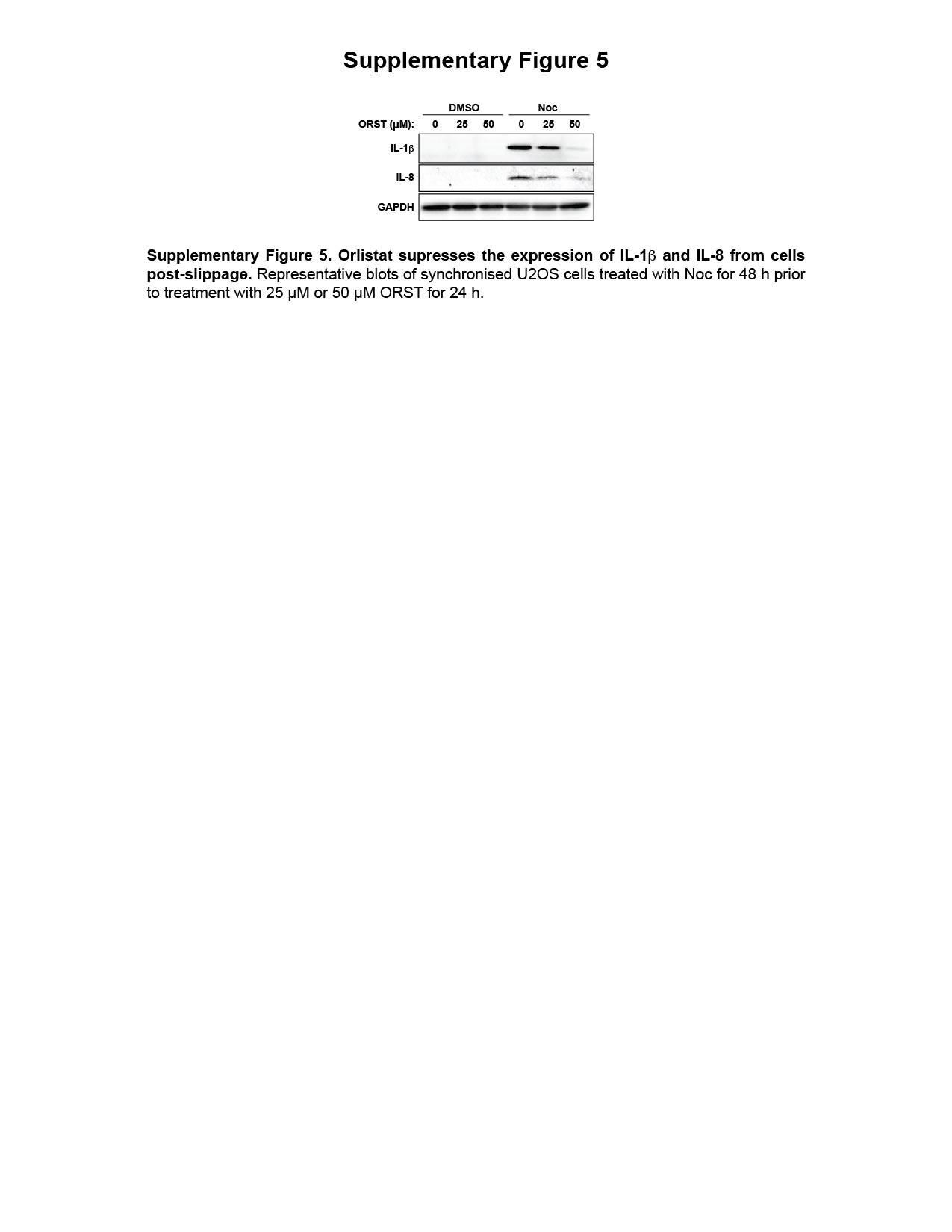

Supplement: Supplementary file 5 — Supplementary Fig S5 [file 41420_2018_127_MOESM5_ESM.jpg]
