# Supplementary material for: Lipid accumulation facilitates mitotic slippage-induced adaptation to anti-mitotic drug treatment
Source: Cell Death Discov. 2018 Nov 27;4:109. doi: 10.1038/s41420-018-0127-5 (PMC6258763; doi:10.1038/s41420-018-0127-5)
Supplement: Supplementary file 6 — Supplementary Table 1 [file 41420_2018_127_MOESM6_ESM.pdf]

Supplementary Table 1

Gene	Fold change	*p*-value	Description
**FABP4**	1.36	0.0071	Fatty acid uptake and transport
**PLIN2**	1.98	0.0002	Lipid droplet-associated protein

Supplementary Table 1. Microarray analysis of fatty acid uptake-related gene expression in post-slippage U2OS cells (nocodazole-treated) versus control (DMSO-treated cells). Accession number: GSE114515.
